# Abrupt skin lesion border cutoff measurement for malignancy detection in dermoscopy images

**DOI:** 10.1186/s12859-016-1221-4

**Published:** 2016-10-06

**Authors:** Sertan Kaya, Mustafa Bayraktar, Sinan Kockara, Mutlu Mete, Tansel Halic, Halle E. Field, Henry K. Wong

**Affiliations:** 1 Department of Computer Science, University of Central Arkansas, Conway, AR USA; 2 Department of Bioinformatics, University of Arkansas-Little Rock, Little Rock, AR 72204 USA; 3 Department of Computer Science and Information Systems, Texas A&M-Commerce, Commerce, TX USA; 4 Department of Dermatology, University of Arkansas for Medical Sciences, Little Rock, AR 72205 USA

## Abstract

**Background:**

Automated skin lesion border examination and analysis techniques have become an important field of research for distinguishing malignant pigmented lesions from benign lesions. An abrupt pigment pattern cutoff at the periphery of a skin lesion is one of the most important dermoscopic features for detection of neoplastic behavior. In current clinical setting, the lesion is divided into a virtual pie with eight sections. Each section is examined by a dermatologist for abrupt cutoff and scored accordingly, which can be tedious and subjective.

**Methods:**

This study introduces a novel approach to objectively quantify abruptness of pigment patterns along the lesion periphery. In the proposed approach, first, the skin lesion border is detected by the density based lesion border detection method. Second, the detected border is gradually scaled through vector operations. Then, along gradually scaled borders, pigment pattern homogeneities are calculated at different scales. Through this process, statistical texture features are extracted. Moreover, different color spaces are examined for the efficacy of texture analysis.

**Results:**

The proposed method has been tested and validated on 100 (31 melanoma, 69 benign) dermoscopy images. Analyzed results indicate that proposed method is efficient on malignancy detection. More specifically, we obtained specificity of 0.96 and sensitivity of 0.86 for malignancy detection in a certain color space. The F-measure, harmonic mean of recall and precision, of the framework is reported as 0.87.

**Conclusions:**

The use of texture homogeneity along the periphery of the lesion border is an effective method to detect malignancy of the skin lesion in dermoscopy images. Among different color spaces tested, RGB color space’s blue color channel is the most informative color channel to detect malignancy for skin lesions. That is followed by YCbCr color spaces Cr channel, and Cr is closely followed by the green color channel of RGB color space.

## Background

Skin cancer is one of the most prevalent cancer types in the United States. The prevalence of skin cancer is increasing dramatically in the United States [1]. Each year the number of patients being diagnosed has raised compared to the previous year. The most common skin cancer type for young adults between 25 and 29 years old is melanoma [1] which can lead to metastatic disease with serious complications from surgery such as scarring, deformity, and death.

Dermoscopy is a method in which pigmented and non-pigmented skin lesions’ features are examined with a handheld device, known as dermatoscope, by health professionals. Dermoscopy is also known as dermatoscopy, skin-surface microscopy, epiluminescence microscopy (ELM) [2, 3]. The ultimate goal of dermoscopy is the early diagnosis of malignant lesions, especially melanomas, by distinguishing them from benign. In order to differentiate malignant skin lesions from benign ones, the abnormal structural features and the borderline of non-pigmented skin lesions need to be taken into account.

Due to the fact that microstructure of the epidermis, the dermoepidermal junction and the papillary dermis are not detectable by the naked eye, dermoscopy is an invaluable asset for diagnosing particularly pigmented skin lesions. According to Vestergaard et al. [4], in the case of the diagnosis of melanoma, the use of dermoscopy provides more accurate results than using clinical evaluation alone by the naked eye.

**Fig. 1:**
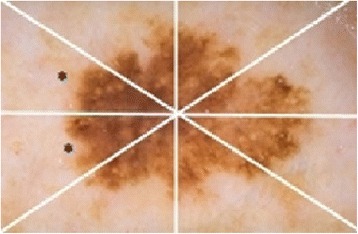
A virtual pie illustrated with *white lines* in a sample lesion, *asterisks* indicate abrupt cutoff in a virtual pie

Potential signs and symptoms of melanoma could be identified by taking the ABCD (Asymmetry Border Color Dermoscopic Features) rule [5] into account during examination of a skin lesion. The ABCD rule assesses geometric and morphologic variables such as asymmetry, color, and border of a given melanocytic lesion [5]. Each of these A,B,C,D features has a preassigned clinical weight factor (e.g. Border’s weight factor is 0.1). B stands for border and it indicates abrupt cutoff in the region of interest (a virtual pie, See Fig. 1). Abrupt cutoff is the region where lesion has sharp circumscription. A virtual pie refers to quarters (pie pieces) of lesion circumference. Figure 1 illustrates a virtual pie and marks (e.g. asterisk) pie pieces with sharp lesion circumscription. For instance B scores 2*0.1 in Fig. 1, since there are two pie pieces marked with abrupt cutoff (e.g. asterisks). A lesion’s B value is calculated if there exists abrupt cutoff at least one quarter of the lesion circumference. Due to applicability of the ABCD rule, it is also recommended for use by clinicians who are not fully trained in dermoscopic observation [6]. Reliability of the ABCD [7] rule has been tested by Nachbar et al. [5]. In this study, it is reported that diagnostic accuracy for melanoma was 80.0 % by using ABCD rule compared to 64.4 % by the naked eye. Assessment of asymmetry, color, differential structure, and border in ABCD rule was used to build a total dermoscopy score (TDS). Each of these features in ABCD contributes to TDS score according to their preassigned clinical weights (e.g. A’s weight is 1.3, B’s weight is 0.1, C’s weight is 0.5, and D’s weight is 0.5). If TDS value is less than 4.75, it is an indicator for lesion being benign. If it is between 4.8 and 5.45, then that lesion is suspicious for malignancy. If TDS score is greater than 5.45, it indicates that the lesion is malignant [7].

A clinical study conducted on 44,258 histopathologically examined skin neoplasms [8] shows that sensitivity in the clinical diagnosis of malignant melanoma is 70.1 %. Even though there have been improvements in the clinical diagnosis of melanoma, melanomas have different clinical accuracies based on their subtypes. For instance, a study by Lin et al. [9] shows that melanoma subtypes of superficial spreading melanomas (SSM), acral lentiginous melanomas (ALM), nodular melanomas (NM), and desmoplastic melanomas (DM) have different clinical diagnosis sensitivity rates. Sensitivity rates of these melanoma subtypes are reported as 77 % for SSM, 73 % for ALM, 41 % for NM, and 21 % for DM.

Moreover, the diagnostic accuracy of a dermatologist is significantly depended on the degree of experience of the examiner. Thus, interobserver variability for diagnosis of pigmented skin lesions amongst dermatologists is an important aspect for clinical diagnostic accuracy. Tan et al. [10] shows up to 0.59 kappa value differences between different dermatologists on the diagnosis of the same cases of melanoma lesions. In the same study [10], interobserver difference reaches up to 0.39 kappa value for benign lesions. Kappa value is a statistical measure for finding inter-rater agreement. For instance, a perfect agreement between two dermatologists on diagnosis of the same cases will result in a kappa value of 1.0, whereas a perfect disagreement on diagnosis of the same cases results a kappa value of 0.0.

One of the criteria for detecting melanoma is abrupt cutoff (abrupt edge). In current clinical practice, in order to detect abrupt cutoff feature for malignancy detection; the lesion is segmented into eight virtual pies (see Fig. 1) in which a dermatologist examines for abrupt cutoff and assigns a score for each of the pie pieces. This process is a manual and a tedious process, over simplified, and it is subjective based on the experience of the dermatologist examining the lesion. To address this challenge, in this study we offer a novel approach to quantify abrupt cutoff along the periphery of the skin lesion border. Instead of analyzing eight virtual pies, we scan whole lesion border’s inner region and quantify each region. To do that, the first step was to accurately define the lesion border.

There are different lesion border detection methods in the literature [11]. We use our density-based lesion detection method (DBLD) [12, 13] for skin lesion border detection. Then Freeman chain code [14] is used to extract extreme border pixels from the detected lesion border. After that, the shrinking (contraction) and the shifting operations are applied to detected border to identify inner border contour. This contour (shadow border) is needed for texture homogeneity analysis. Shadow border refers to a contracted border of the lesion. Shadow borders are found by using vector operations and dynamic scaling operations. Then, along shadow borders, pigment patterns’ statistical texture measures and statistical texture features, especially homogeneity, are computed in various scales in various color spaces. Different color models and color channels are used to investigate which color model is more effective in dermoscopy image analysis. Reader is referred to [15] for details on analysis of color models and color channels on biomedical image processing.

Textures in an image refer to visual patterns with different characteristics such as color, brightness, slope, size, uniformity, roughness, regularity, randomness, granulation, fineness, and coarseness etc. There are mainly structural, statistical, model-based, and transform methods for texture analysis [16]. Structural methods define textures in micro and macrostructural level and there is no clear distinction between them. Structural methods fit better for texture synthesis than texture analysis. Statistical methods attempt to capture non-deterministic properties of an image. More specifically, statistical methods capture distributions and relationships of grey levels. The most popular statistical texture analysis is based on co-occurence matrix. Three of the most common statistical texture analysis methods are based on the Gibbs random field, the Gaussian random field, and the Markov random field (MRF) models [17, 18]. Model based methods use fractals and stochastic models; however, estimation of parameters in both fractal and stochastic based models is a major bottleneck. Transform based methods use different transformation functions such as Fourier, Gabor, and wavelet. In this group of texture analysis methods, image is represented in a different coordinate space. In transform based methods, Fourier poorly performs because after the transform, spatial localization information is lost. On the other hand, Gabor provides better spatial localization; however, it needs multiple filter resolutions to capture spatial structures such as natural textures. This makes the use of Gabor impractical for most cases. Wavelet, on the other hand, represents textures at varying spatial resolutions with varying wavelet functions. However, wavelet transform is not translation invariant.

In a recent study [19] investigators used 95 texture descriptors for melanoma detection in dermoscopy images. However, they obtained up to 83 % specificity and 53 % sensitivity in grayscale images for melanoma detection. In this study, they also found out that image’s uniformity is the best descriptor with standalone 70 % accuracy.

## Methods

### Dermoscopy image analysis

#### Preprocessing

**Fig. 2:**
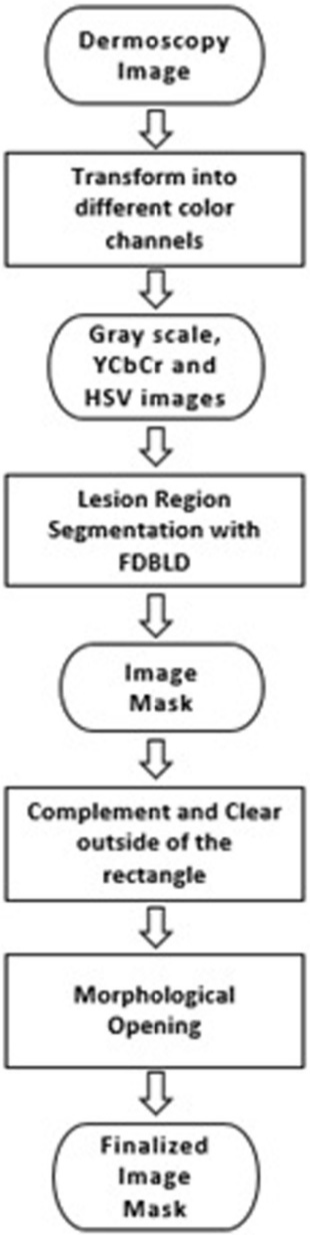
Preprocessing flow scheme

We describe essential image processing operations to make the dermoscopy image and its mask (whole lesion; represented as black pixels in the mask) ready for the further steps. These operations include: color space transformations, lesion border detection with density-based lesion detection method [9], complement operation, clearing region remains outside of the rectangle (region of interest), and morphological opening operations. The flow diagram of preprocessing phase is shown in Fig. 2.

**Fig. 3:**
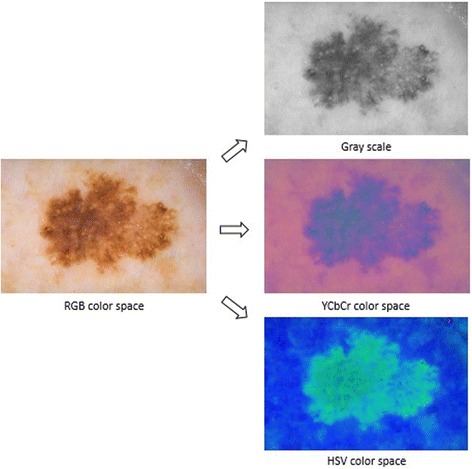
Color Space Conversion

**Fig. 4:**
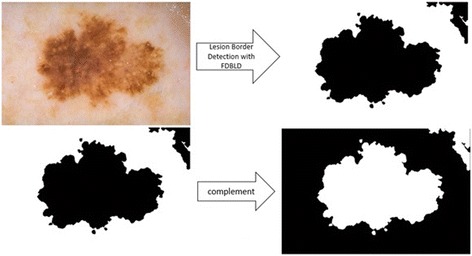
Preprocessing operations: Lesion Border Detection with DBLD and Complement

In the color transformation phase, RGB color space of dermoscopy image is transformed into different color spaces; gray scale, YCbCr, and HSV color spaces respectively (See Fig. 3). In the next step, in order to obtain the mask from dermoscopy image, density-based lesion detection method [12, 13] is used. These steps are illustrated in Fig. 4.

Moreover, since the mask is a negative version of a regular standardized mask, the complement operation is applied to the mask to generate a standardized mask. Some of the images’ masks include noisy pixels other than the region of interest. Thus, we clear the region outside of the region of interest (out of the rectangle that encapsulates lesion). For finding this rectangular region we use following equations which incorporate percentage clipping parameter from left, right, top, and bottom from the mask.

1.1 $$ Left\  Boundary=1+\frac{w}{2\ast \frac{percentage\_ of\_ edges\_to\_ clip}{100}\ } $$

1.2 $$ Right\  Boundary=w-\frac{w}{2\ast \frac{percentage\_ of\_ edges\_to\_ clip}{100}\ } $$

1.3 $$ Top\  Boundary=1+\frac{h}{2\ast \frac{percentage\_ of\_ edges\_to\_ clip}{100}\ } $$

1.4 $$ Bottom\  Boundary=h-\frac{h}{2\ast \frac{percentage\_ of\_ edges\_to\_ clip}{100}\ } $$

**Fig. 5:**
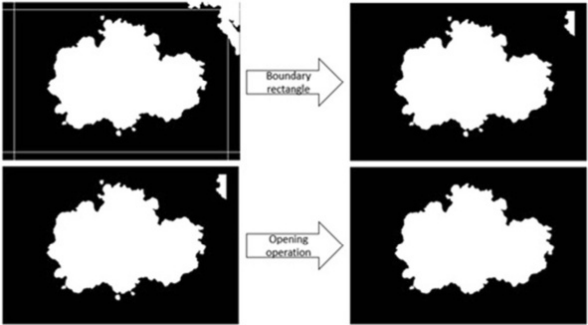
Preprocessing operations: Clear outside of the rectangle and Opening operations

In the same methodology, the right, top, and bottom boundaries’ pixel locations are determined by the equations above. Percentage of edges to clip parameter is empirically determined for our dataset. This parameter is based on the observations on dermoscopy images that lesion is always on the center of dermoscopy images. Thus, the boundary rectangle tightly encapsulating the lesion can be drawn around the region as seen in Fig. 5. Region remains outside this rectangle is discarded from further processing. This provides two-fold benefit. First, this reduces overall computation time; second, removes noise and eventually increases accuracy of the results. Noise refers to clustered regions that are separated from the lesion.

The final step of the preprocessing phase is morphological opening operation [20]. Morphological opening operation aims to even further remove the leftover noisy pixels from the mask. Opening simply removes small objects from the foreground of the image and puts them back into the background. The mathematics behind morphological opening operation is described in the following equation.

1.5 $$ A\circ B=\left(A\ominus B\right)\oplus B $$

where A denotes an image, B refers to a structuring element and two operation symbols are an erosion and a dilation respectively. Dilation is a morphologic operation which causes objects to dilate or grow in size; whereas erosion causes objects to shrink. The amount of growth or shrinkage depends on the choice of the structuring element. Structural element differentiates dilation/erosion from classical low pass filter. Two of the well-known common structuring elements (given a Cartesian grid) are the 4-connected and 8-connected sets, N_4_ and N_8_ respectively. Morphological opening operation is applied to the mask. This morphological opening after edge clipping operation (given in Eqs. from 1.1 to 1.4 illustrated in Fig. 5. Noises are caused by artifacts such as hair, water etc. in dermoscopy images. When image is segmented by DBLD, some of these artifacts are also clustered as disconnected or connected parts of the lesion. In order to remove these regions from further processing, we use a commonality for every dermoscopy image; lesion exists approximately at the center of the image. The largest cluster including the vicinity of the center of the image is our region of interest; thus, other clusters are marked as noise and discarded from further processing. In order to discard the noisy regions, edge clipping and morphological operations are used.

#### Boundary pixels detection

**Fig. 6:**
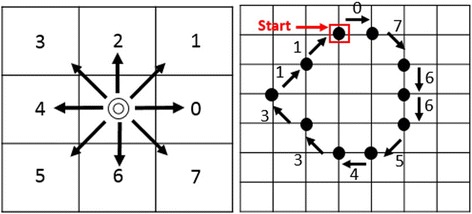
**a** Rectangular-grid and **b** Digital boundary on grid

The chain code was first proposed by Herbert Freeman [14] and, thus, it is also known as Freeman code or Freeman Chain code in the literature. He proposed that an arbitrary geometric curve could be represented by utilizing a rectangular-grid. Moreover, the idea behind the chain code is to obtain a way to identify a binary object representation by encoding its boundary. In a continuous curve, since consecutive points are adjacent to each other, each point is dependent on the previous one. For that reason, it causes a limitation for the next point’s location. Therefore, the next point can only have 8 possible locations which are a sequence of numbers from 0 to 7 in the rectangular-grid. Each number refers to a transition direction in between two consecutive points. As it is seen in the rectangular-grid (see Fig. 6), the increase of numbers progresses in the counter-clockwise direction.

Interpretation of the numbers is that one walks along the pixels on the object’s boundary from starting and ending at the same pixel. For instance, for the given digital boundary in Fig. 6, when one walks around the shape in the clock-wise direction, it would result in a number sequence such as {0,7,6,6,5,4,3,3,1,1}. This sequence simply is referred to as an encoding form of the boundary.

**Fig. 7:**
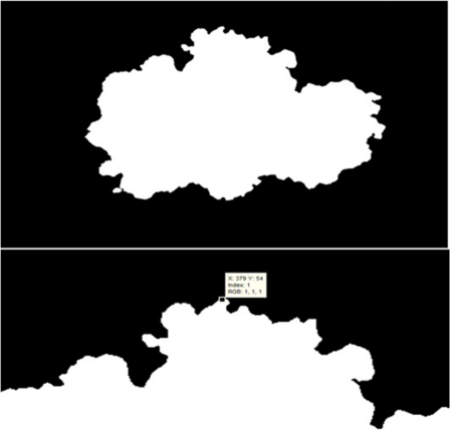
**a** The finalized dermoscopy image mask **b** The starting pixel

The goal of this stage is to determine extreme pixels at the lesion border. The idea behind this approach is to first scan through the entire pixels and record the ones which belong to the foreground. Then, find the minimum among all rows (vertical direction of the image mask) pixels. With the help of this minimum row value, the lowest value among all column pixels is detected in the image mask. By doing so, this approach gives us the row and column of the starting pixel for the chain code. Once starting pixel for the chain code is determined, the chain code generates a list of clockwise or counter-clockwise adjacent pixels. The starting pixel is shown in Fig. 6b. By applying the chain code, the boundary of the region in a dermoscopy image is captured as depicted in Fig. 7.

### Feature extraction

For feature extraction, we developed three different approaches: texture feature extraction along the lesion border (detailed in section 2.1), along the inner border which is found by utilizing vector operations towards the center of the lesion’s mass (detailed in section 2.2), and along the inner aligned/shifted border by utilizing a scaling factor (detailed in section 2.3) respectively. From these three different methods, we extract statistical measures and statistical texture features of dermoscopy images and compare each method’s accuracy for malignancy detection. Statistical measures that are considered here are mean and standard deviation [21], whereas Gray Level Co-occurrance Matrix (GLCM) [20] is described as a texture descriptor in the texture analysis.

The gray-level co-occurrence matrix (GLCM) [20] is a statistical method that scrutinizes texture characteristics that rely on the spatial relationship between pixels. GLCM is also referred to as the gray-level spatial dependence matrix and co-occurrence distribution. The texture of an image can be represented with GLCM functions where specific values of pairs of pixels are computed and spatial relationship that arises in the image are represented by the GLCM. Thus, statistical measures can be extracted from the GLCM matrix. Mathematically, the gray-level co-occurrence matrix (C, as given in below Eq. 2.1) can be described over an image in which co-occurrence distribution parameters are illustrated for specific offset values.

2.1 $$ {C}_{\Delta x,\Delta y}\left(i,j\right)={\displaystyle {\sum}_{p=1}^n{\displaystyle {\sum}_{q=1}^m\left\{\begin{array}{c}\hfill 1,\  if\ I\left(p,q\right)=i\  and\ I\left(p+\Delta x,\ q+\Delta y\right)=j\ \hfill \\ {}\hfill 0,\kern0.75em  otherwise\hfill \end{array}\right.}} $$

where C is the co-occurrence matrix, I is the image with nxm size, i and j are the image intensity values, *p and q* are the spatial coordinates in the image, and lastly (∆*x*, ∆*y*) is an offset parameter which is the function of the direction *θ* and the distance *d*. The co-occurrence matrix is susceptible to rotation due to offset parameters. In order to create the GLCM, the gray co-matrix function [22] is taken into account which constructs the gray-level co-occurrence matrix (GLCM). GLCM is a square matrix where its columns and rows are equal to the number of gray levels in the image. The matrix element *C*_Δ*x*,Δ*y*_(*i*, *j*) corresponds to a relative frequency of two pixels with intensity values i and j respectively. Moreover, these pixels are separated by a pixel distance (*x*, ∆*y*).

However, other spatial relationships can be defined among the two pixels such as ∆x = 2, ∆y = 1. The sum of the frequency occurrence of pixel intensity value i with respect to the pixel intensity value j based on the particular spatial relationship which builds up the basis of each element (i,j) in the GLCM matrix. The size of the GLCM refers to the number of gray levels in the image.

Moreover, numerous statistical features can be acquired by utilizing the GLCM matrix. Homogeneity measures how similar the gray levels are in the spatial distribution of the image. Homogeneity of an MxN image can be expressed with the equation below.

2.2 $$ {\displaystyle {\sum}_{i=1}^m{\displaystyle {\sum}_{j=1}^n\frac{GLCM\ \left(i,j\right)}{\left(M-\Delta \mathrm{x}\right)\left(N-\Delta \mathrm{y}\right)}}} $$

In Eq. (2.2), M denotes number of pixels in the vertical direction and N denotes number of pixels in the horizontal direction of an image.

In most cases, the spatial relationship between two pixels is described as the pixel of interest which is the right adjacent pixel of the current pixel. This means that ∆x = 1, ∆y = 0. For instance, if ∆x = 1, ∆y = 0 is the spatial relationship between two pixels of an exemplary 5 × 5 image as given below:

IMAGE (5 × 5 with given intensity values)

$$ \begin{array}{ccccc}\hfill 0\hfill & \hfill 1\hfill & \hfill 1\hfill & \hfill 3\hfill & \hfill 0\hfill \\ {}\hfill 0\hfill & \hfill 1\hfill & \hfill 2\hfill & \hfill 3\hfill & \hfill 0\hfill \\ {}\hfill 1\hfill & \hfill 2\hfill & \hfill 3\hfill & \hfill 2\hfill & \hfill 0\hfill \\ {}\hfill 1\hfill & \hfill 1\hfill & \hfill 2\hfill & \hfill 2\hfill & \hfill 0\hfill \end{array} $$

Then the corresponding co-occurrence matrix will be a 4 × 4 matrix as given below:

Co-occurence Matrix (4 × 4; there exists 4 different intensities in the image)

$$ \begin{array}{cccc}\hfill 0/20\hfill & \hfill 2/20\hfill & \hfill 0/20\hfill & \hfill 0/20\hfill \\ {}\hfill 0/20\hfill & \hfill 2/20\hfill & \hfill 3/20\hfill & \hfill 1/20\hfill \\ {}\hfill 2/20\hfill & \hfill 0/20\hfill & \hfill 1/20\hfill & \hfill 2/20\hfill \\ {}\hfill 2/20\hfill & \hfill 0/20\hfill & \hfill 1/20\hfill & \hfill 0/20\hfill \end{array} $$

For instance first (0,0) element of co-occurrence matrix corresponds to the frequency of neighboring two pixels in entire image with both pixels having intensity value of 0 with the defined spatial neighborhood relationship of ∆x = 1, ∆y = 0. This relationship corresponds to the number of times that 0 intensity pixel’s right next neighbor pixel also has intensity value of 0. This relation never exists in the sample 5x5 image; thus, co-coccurence matrix’s (0,0) element’s value will be 0. The term 1/20 comes from (M-∆x)*(N-∆y) in Eq. 2.2. For the given 5x5 image example, M = 5, N = 5, ∆x = 1, and ∆y = 0.

#### 1^st^ method: texture feature extraction on the boundary

**Fig. 8:**
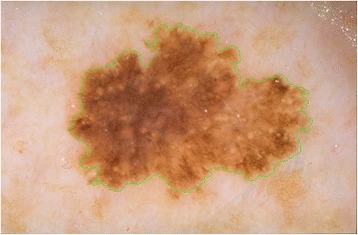
The detected boundary

**Fig. 9:**
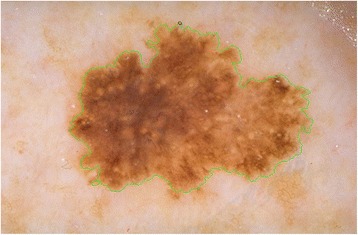
The first *circle* on the boundary

**Fig. 10:**
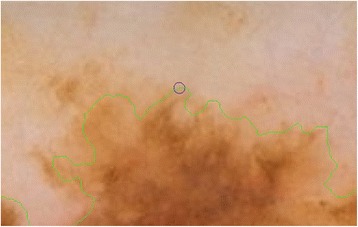
Zoom in the *circle*

The goal of this approach is to extract the features mentioned in previous sections from the border of dermoscopy images by employing circular masks at different scales. In order to carry out utilizing circular masks on the border of dermoscopy images, first, the radius and the coordinates of the center of the circle must be determined. Hence, for the first circle’s center, we pick a pixel at the lesion border which is the upmost pixel. This is illustrated in Figs. 8, 9, and 10.

**Fig. 11:**
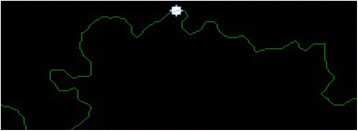
The Circular mask

The next step is the feature extraction from where the circular mask, which is illustrated in Fig. 11, is located. Feature extraction is a computation of statistical features of a particular region. In our case, in order to obtain the region from the image, an element by element matrix multiplication is performed in between a color channel of the image and the circular mask. This is expressed in the following equation.

2.3 $$ Circular\_ region= Image\_ channel.\ast Circular\_ mask $$

**Fig. 12:**
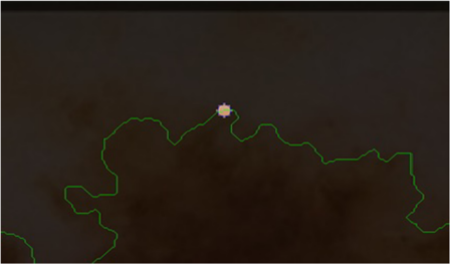
The Circular region mapped on an actual dermoscopy image

The result of this element by element matrix multiplication operation is shown in Fig. 12. The obtained *Circular_region* now can be taken into account to accomplish feature extraction operations which are mean, standard deviation and homogeneity with the help of GLCM matrix.

In essence, this feature extraction cycle is simply performed in a particular image channel for each circle along the lesion border. This process continuous for each circle along the lesion border until the cycle is completed. Cycle is completed when process returns back to where it is started (upmost pixel). This method is employed on all 100 images with varying radius of the circles. Algorithm 1 summarizes this entire process below.
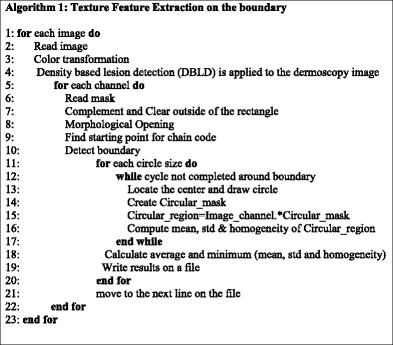


#### 2^nd^ method: texture feature extraction on the inner-shifted by vector scaling

The purpose of this method, which is also summarized in the Algorithm 2, is to describe how the vector scaling operation and polygon techniques are achieved in order to identify the intersecting polygon region and extract them from a dermoscopy image. First and foremost, the centroid of the segmented skin lesion is located and marked on the finalized dermoscopy image mask. For this method, a vector is defined in between the starting point on the boundary of the mask and the centroid of the segmented skin lesion. Then, the unit vector is computed from this vector by utilizing Eq. (2.4). Moreover, the unit vector is used to perform a vector operation, namely vector scaling, and to shift boundary points towards to the centroid of the segmented skin lesion. This vector scaling and the shifting operations are shown with the Eqs. (2.5) and (2.6) respectively. For the given equation,

2.4 $$ \overrightarrow{u}=\frac{centroid\left(x,y\right) - boundary\left(x,y\right)}{\left\Vert centroid\left(x,y\right) - boundary\left(x,y\right)\right\Vert } $$

where *centroid* (*x, y*) is the center of mass of the skin lesion, *boundary* (*x, y*) is the starting point (upmost pixel) on the boundary of skin lesion and finally $\overrightarrow{u}$ is the unit vector at the boundary directed towards the center. The vector scaling is given by

**Fig. 13:**
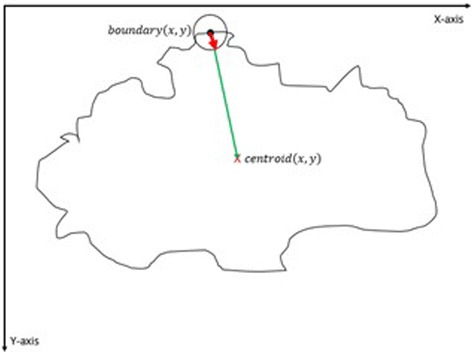
The vector and the unit vector in between centroid(x,y) and boundary(x,y)

**Fig. 14:**
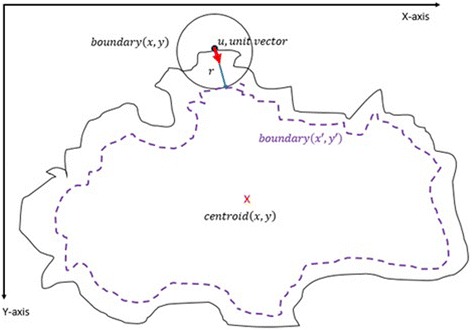
The shifted boundary(x', y')

2.5 $$ \overrightarrow{u}\cdot r=u\ast r=\left({u}_x\ast r,\ {u}_y\ast r\right) $$

where $\overrightarrow{u}$ is the unit vector directed towards the center and r is the radius (a scalar). Furthermore, in order to perform shifting operation to the boundary, simply, the result of a vector scaling operation, which is $\overrightarrow{u}\cdot r$, is added to the original boundary coordinates, *boundary*(*x, y*). Equation (2.6) basically shows how to perform shifting operation. For the given equation,

2.6 $$ boundary\left(x^{\prime },y^{\prime}\right)=\left( boundary(x) + {u}_x\cdot r,\  boundary(y) + {u}_y\cdot r\ \right) $$

where *r* denotes radii, *u*_*x*_ and *u*_*y*_ refer to x and y coordinates of u unit vector. Figure 13 illustrates how to create the vector between *centroid*(*x, y*) and *boundary*(*x, y*) and finding a unit vector along that direction. After applying shifting operation (shrinks/contracts boundary towards centroid) to each point of the original boundary, it would create a boundary which is illustrated as dotted boundary in Fig. 14.

**Fig. 15:**
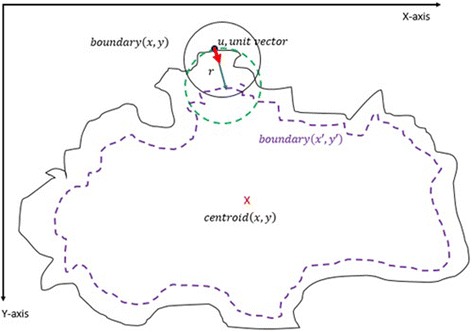
The intersecting polygon regions on both borders

**Fig. 16:**
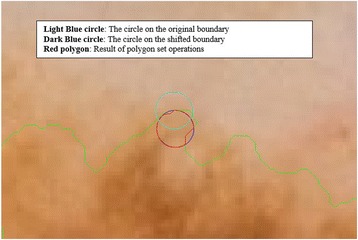
The red polygon is the resulting polygon after set operations

**Fig. 17:**
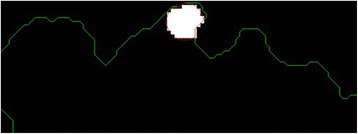
The mask of intersecting polygon region (*white color*)

The next step is to investigate texture features of the circular regions along the contracted new boundary. As it is seen in Fig. 14, there are two boundaries; actual lesion boundary and contracted boundary where in-between distance of these boundaries are always r. We find the texture homogeneities and statistics between these two boundaries in circular regions whose centers are placed on the inner boundary. Those circular regions are equally distanced from each other. These circles distances are set as 3/2*r (which signifies that r is scaled in 1.5 fold) and this is measured by using the chain code. For instance if r is 2 then next center will be 3 pixels apart in the chain code. After locating all circular regions’ centers on the inner boundary, we apply polygon intersection operation between these circular regions and the actual boundary for each circular region. This helps us to remove noisy data coming from pixels out of the actual boundary, which reduces the automated diagnosis accuracy. It is noted that the intersecting region must be inside of the outer boundary, the reason is that outside of the boundary is considered as noise or outliers that is not incorporated into the intersecting region. Since these regions insert noises into the region of interest, it reduces the accuracy of the results. An exemplary polygon intersection operation between a circular region and the boundary is illustrated in Figs. 15 and 16. Mask of the intersecting polygon is generated for further steps of the work. The mask is illustrated in Fig. 17.

**Fig. 18:**
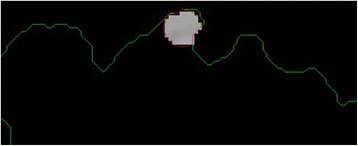
The extracted region of the intersecting polygon region mapped on an actual dermoscopy image

In the last step of this method, in order to extract the intersecting polygon region, an element by element matrix multiplication operation is performed between actual image and the intersecting polygon mask. This helps us to extract texture information of the corresponding region of interest which are the pixels located at the coordinates of the mask’s elements (e.g. pixels). The extracted region is illustrated in Fig. 18. This matrix operation’s equation is described as following:

2.7 $$ Polygon\_ region= Image\_ channel.\ast Polygon\_ mask $$

**Fig. 19:**
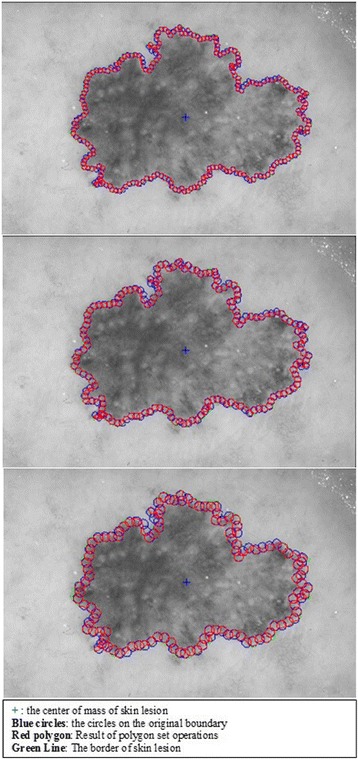
The intersecting *polygon circle* with radius size (**a**) 5 (**b**) 7 (**c**) 10

**Fig. 20:**
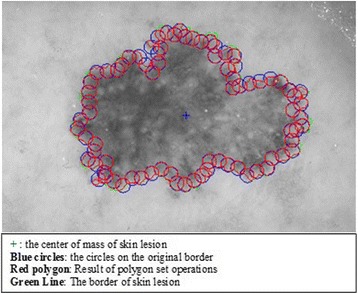
The intersecting *polygon circle* with radius size 15

Then, the statistical texture measures (mean and standard deviation) and the statistical texture features, particularly homogeneity features, are computed over this polygon (e.g. mask and image intersecting pixels). Figures 19 and 20 illustrate the intersecting polygon feature extraction at different scales (different radii).

**Fig. 21:**
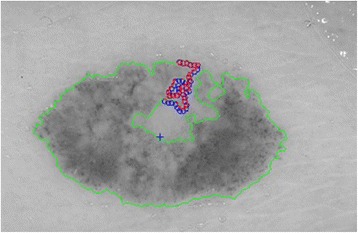
The failing case of the dermoscopy image


Additionally, there are some images which include certain characteristics where this method (e.g. shrinking border towards the center of mass) simply fails while determining the intersecting polygon region. For instance, a sample case is shown in Fig. 21 where the extracted region is simply outside of the boundary. This is due to the imbalance of the mask where the unit vector’s definition becomes invalid. Another reason is that when the border is contracted towards the center of mass, many points are mapped to a single point (there is not necessarily unique one-to-one mapping). That’s the reason why we developed a 3^rd^ method to overcome these deficiencies in the 2^nd^ method.

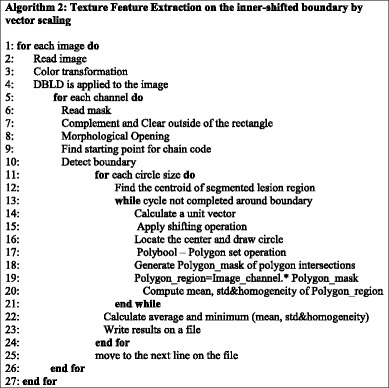


#### 3^rd^ method: texture feature extraction on the inner-shifted by dynamic scaling

The aim of this method is to scale down the boundary by means of a dynamic scaling approach, so that the original boundary would be shifted to the inside and with the help of circular regions, the polygon intersection is determined, and features are calculated over the intersecting polygon region.

**Fig. 22:**
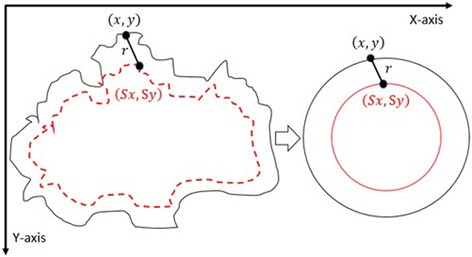
From irregular to perfect *circle*

First of all, the centroid of the segmented lesion is located and marked on the finalized dermoscopy image mask. The novelty of this method is to explore how to obtain scaling factor. The proposed approach is that the segmented lesion and the scaled region are assumed as perfect circles in a coordinate system. This assumption is shown in Fig. 22 on the right. Under this assumption, the radius is known and the distance between the starting point *(x,y)* and the scaled point *(sx,sy)* can be written by means of the following Eq. (2.8).

2.8 $$ {\left(sx-x\right)}^2 + {\left(sy-y\right)}^2={r}^2 $$

2.9 $$ {(sx)}^2+{x}^2-2s{x}^2 + {(sy)}^2+{y}^2-2s{y}^2={r}^2 $$

2.10 $$ {x}^2\left({s}^2+1-2s\right)+{y}^2\left({s}^2+1-2s\right)={r}^2 $$

2.11 $$ {\left(s-1\right)}^2\left({x}^2+{y}^2\right)={r}^2 $$

2.12 $$ {\left(s-1\right)}^2=\frac{r^2}{\left({x}^2+{y}^2\right)} $$

2.13 $$ s-1=\pm \sqrt{\frac{r^2}{\left({x}^2+{y}^2\right)}} $$

2.14 $$ s=1-\sqrt{\frac{r^2}{\left({x}^2+{y}^2\right)}} $$

By deriving the distance Eq. (2.8), the scaling factor becomes (2.13) and the negative one (2.14) is taken into account due to the fact that the operation would be a scale down operation (taking the inner border). Next step is applying this scaling factor to each and every pixel at the lesion border.

Scaling [23] is a transformation where the image size gets bigger or smaller. We are interested in scaling down the original image’s lesion boundary since out of lesion boundary is considered as noise/outlier (e.g. reduces diagnostic accuracy). This transformation is expressed with the Eqs. (2.15) and (2.16).

2.15 $$ x^{\prime }={s}_xx $$

2.16 $$ y^{\prime }={s}_yy $$

where x’ is a scaled down x coordinate and y’ is a scaled down y coordinate of a corresponding boundary pixel’s x and y coordinates respectively, and *s*_*x*_ and *s*_*y*_ are scaling factors along x and y axes for 2D.

This can be also written in a matrix form with homogenous coordinates where last column represents translation (used for aligning centers of actual lesion border and newly produced inner lesion border) as follows.

2.17 $$ \left[\begin{array}{c}\hfill x^{\prime}\hfill \\ {}\hfill y^{\prime}\hfill \\ {}\hfill 1\hfill \end{array}\right]=\left[\begin{array}{ccc}\hfill s\hfill & \hfill 0\hfill & \hfill dx\hfill \\ {}\hfill 0\hfill & \hfill s\hfill & \hfill dy\hfill \\ {}\hfill 0\hfill & \hfill 0\hfill & \hfill 1\hfill \end{array}\right]\left[\begin{array}{c}\hfill x\hfill \\ {}\hfill y\hfill \\ {}\hfill 1\hfill \end{array}\right] $$

**Fig. 23:**
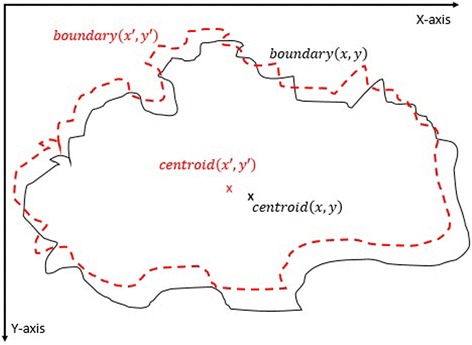
The original and scale down boundary

**Fig. 24:**
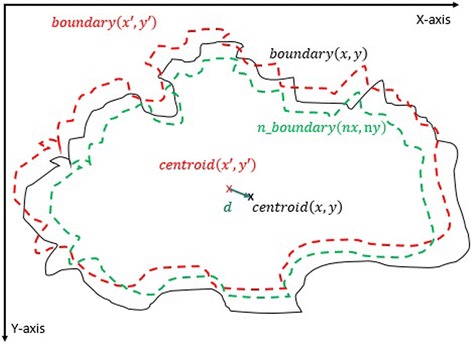
The distance vector and the inner-shifted boundary

After obtaining the scale factor, the new boundary coordinates *boundary*(*x* ′, *y* ′) can be calculated by using Eq. (2.17). This is illustrated in Fig. 23. Since the goal is to extract the regions inside the original boundary, the new *boundary*(*x* ′, *y* ′) has to be shifted (translation last column in transformation matrix in Eq. 2.17) from new center to the old boundary’s center. For that reason, a difference vector $\overrightarrow{d}=\left\{dx, dy\right\}$ is defined in between the centroid of two boundaries which is demonstrated in Fig. 24. This vector is also expressed in the following equation.

2.18 $$ \overrightarrow{d}= centroid\left(x,y\right) - centroid\left(x^{\prime },y^{\prime}\right) $$

In the next step, the $\overrightarrow{d}$ vector is utilized to locate the inner shifted boundary with the help of the Eq. 2.19. The inner shifted boundary *n*_*boundary*(*nx*, *ny*) is displayed in Fig. 24.

2.19 $$ n\_ boundary\left(nx,ny\right)=\left( boundary\left({x}^{\prime}\right)+{d}_x, boundary\left({y}^{\prime}\right)+{d}_y\right) $$

**Fig. 25:**
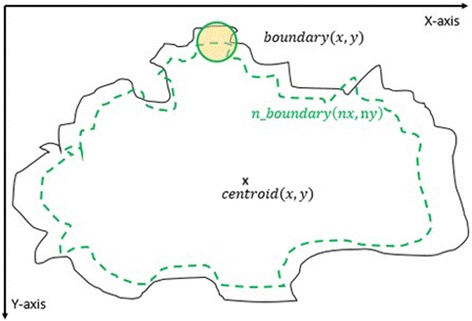
The intersecting polygon region

**Fig. 26:**
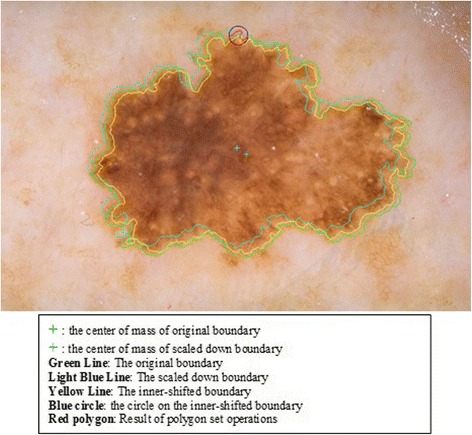
The result of polygon set operations

Moreover, the circle can be located and drawn on the inner-shifted boundary as depicted in Fig. 25. Now, in order to figure out the intersecting polygon regions among two boundaries and the circle, the polygon set operations are applied to all of the circular regions. The outcome of polygon set operations is given in Fig. 26 which is the intersecting polygon region.

**Fig. 27:**
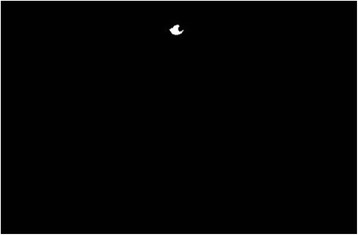
The mask obtained by polymask operation

After having the intersecting polygon region, the mask of the intersecting polygon region is obtained with the help of polymask operations. The resulted mask is shown in Fig. 27.

Moreover, the region extraction operation is carried out in between the image channel and polymask. This is essentially element by element matrix multiplication that can be computed with the Eq. 2.20.

2.20 $$ Polygon\_ region= Image\_ channel.\ast Polygon\_ mask $$

**Fig. 28:**
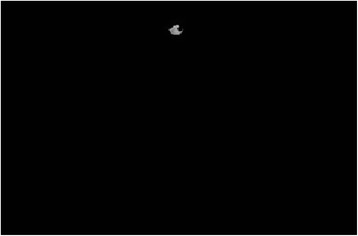
The extracted region by matrix multiplication mapped on the dermoscopy image

**Fig. 29:**
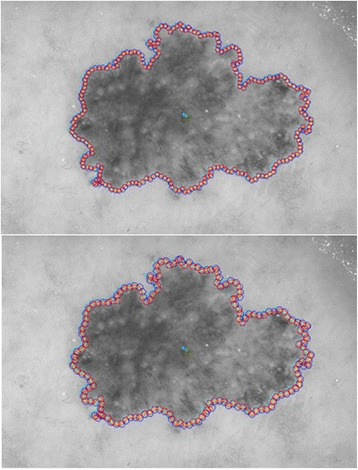
The intersecting *polygon circle* with (**a**) radius size 5 (**b**) radius size 7

Finally, statistical measures (mean and standard deviation) and statistical texture feature, particularly, homogeneity, are calculated over these masks. Intersecting polygon feature extraction is shown at different scales (different radii) by computing features in Figs. 28 and 29.

All these processes are carried out on dermoscopy images in our dataset by considering 10 different color channels along with different scales. In brief, Algorithm 3 simply summarizes all steps mentioned in this section.
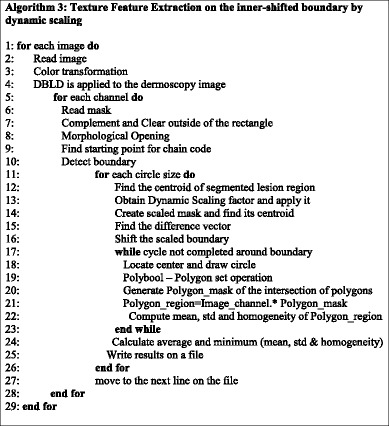


### Experiments and data analysis

A set of 100 dermoscopy images obtained from the Edra Interactive Atlas of Dermoscopy [24] is used as a test bed to perform experiments and validate the proposed approach. These dermoscopy images are 24-bit RGB color images with dimensions ranging from 577 × 397 pixels to 1921 × 1285 pixels.

Initially, the structure of features is created based on the following variations:

- 100 Dermoscopy images
- Color spaces and channels: 10 base colors (Gray, Red, Green, Blue, Y, Cb, Cr, Hue, Saturation, Value)
- Circle radius size: 4 different scale (5, 7, 10, 15)
- Average and Minimum:
  - ○ Homogeneity,
  - ○ Mean
  - ○ Standard deviation

A sample of features is illustrated in Table 1. In each circle’s radius size; average homogeneity, minimum homogeneity, average mean, minimum mean, average standard deviation, and minimum standard deviation are described, and there are 4 different circle radius sizes under red color channel of RGB color space. Moreover, each row also refers to a dermoscopy image and there are 6 rows for the given sample table.

**Table 1:** The structure of 6 sample features in red color channel of RGB color space

Red channel					
Ave. Homogeneity	Min Homogeneity	Ave. Mean	Min Mean	Ave. Std	Min Std
Circle r = 5					
0.917462178	0	0.646102982	0.388763198	0.037210395	0.011219957
0.95077049	0.729166667	0.814483693	0.68583878	0.020879577	0.006814895
0.958515455	0.8	0.739801882	0.620409982	0.020998995	0.007895374
0.982178346	0.758064516	0.73183427	0.58650519	0.01840594	0.005266276
0.827557372	0.678571429	0.75861334	0.657806826	0.05960707	0.030835966
0.864271752	0.718954248	0.728798027	0.621372549	0.049980381	0.029246741
Circle r = 7					
0.915465117	0.836336336	0.632675843	0.430287115	0.047567	0.018876
0.945919765	0.814285714	0.807526368	0.664418938	0.0267441	0.006586
0.962351373	0.851351351	0.733075024	0.624977919	0.0252326	0.011284
0.981805885	0.860655738	0.727046308	0.621084798	0.0228896	0.008088
0.83392547	0.752427184	0.749178241	0.669057867	0.0636916	0.03711
0.867737825	0.743902439	0.724178282	0.59988578	0.0519912	0.03114
Circle r = 10					
0.917726394	0.851851852	0.6125184	0.3844201	0.0584022	0.022342
0.940874749	0.869369369	0.7945501	0.6844033	0.035574	0.012485
0.964213332	0.89	0.7227319	0.6172549	0.0319608	0.015831
0.981313393	0.895061728	0.7206711	0.6165988	0.0281256	0.010769
0.83617572	0.776995305	0.7377832	0.6298727	0.0683642	0.042108
0.869423377	0.786195286	0.7208185	0.5804095	0.0534239	0.037574
Circle r = 15					
0.922607817	0.879204893	0.580591	0.438253	0.0750966	0.028403
0.938572886	0.890572391	0.7760676	0.65931	0.0480889	0.016715
0.966216948	0.910967344	0.7085378	0.599849	0.0403343	0.02023
0.975049182	0.917307692	0.7106661	0.640318	0.03465	0.01445
0.840957946	0.802281369	0.7217243	0.633724	0.0756031	0.051994
0.870191024	0.803108808	0.7173696	0.631668	0.0561643	0.040177

Radii sizes determined through trials and errors from dermatologist drawn lesion borders. We realized that homogeneity reduces drastically right at the lesion border. Thus, starting from radius size 2, we increased the radius size by one and tried to find out whether that radius size capturing drastic homogeneity change or not. We continued increasing the size of radius up until the point that it no longer captures homogeneity of an abrupt change at the border. We chose 4 different radii sizes since they captured sudden homogeneity changes at the lesion border. For instance, results obtained for radius size 4 was almost same with having radius size 5 or 6. Thus, we picked only one of them, radius 5. Since lesion border cutoff happens close to the lesion border, for radius sizes >15 we observed that these cuttoffs are missed. Thus, we stopped at radius size 15 pixels.

One of the major preparation steps to make the features ready is to normalize all features in the range of [−1, 1]. As described above, there are 10 distinct base colors and also each circle radius has 6 unique texture feature types, and finally 4 different scales are considered for this experiment. All these make 240 total number of features per a single dermoscopy image. We extract all these features for 100 dermoscopy images. With these in mind, the challenging question is which features are more significant or in other words, which features will yield better result when the classification experiment is performed by Support Vector Machine (SVM) [25] classifier. Hence, in order to select significant features among all, SVM Recursive Feature Elimination (SVM RFE) [26] algorithm, which iteratively works backward from the given set of features, is employed. In each cycle, it essentially sorts the features according to their weights in the SVM classifier by discarding the features that have lower weight. Once the SVM RFE is applied to all features, it basically yields an order, based on rank (R) and sorts these features accordingly. The higher value R has, the more significant the feature is. The color based sorting is illustrated in the Table 1 along with individual rank (R) score. After generating the ranking of these features, the most informative color-based feature is selected in which each color channel (total of 24 channels) for different color spaces is ranked with the following equation,

3.1 $$ {T}^C={\displaystyle {\sum}_{k=1}^{24}{R}_k,\kern0.75em R\  of\ C} $$

where k is the feature number and R is the ranking value. The Eq. (3.1) will generate a total score for each color channel by summing up R in each color set. Ranking of total score is shown in the Table 2. What can be interpreted from Table 2 is that the sum of the rank of all Blue-based features is 3910 which ranks top in the list. The second best color is Cr, and Green is the third one. Thus, two sets of features, which are listed below, are considered to perform classification experiments.

- Blue, Cr, Green (Top three features according to the rank result)
- Blue, Cr, Green and Fractals by Kockara et al. [27].

**Table 2:** The individual color sorting based upon the rank of each color

Color	Score
Blue	3910
Cr	3293
Green	3237
Y	2904
Cb	2796
Gra	2698
Sat	2647
Hue	2581
V	2428
Red	2426

As seen from scoring results in Table 2, blue color channel has scored the highest for detecting malignancy in dermoscopy images. It is followed by the green channel from RGB color space. This indicates that malignant lesions are more distinctive in blue. There are some studies investigating effects of phototherapy in visible blue and green light that may enlighten why blue channel is more informative for malignancy detection.

Phototherapy with visible light (specifically blue light that ranges 430–490 nm) has been commonly used as a treatment for certain skin diseases such as acne treatment and psoriasis [28, 29]. Red light penetrates deeper in tissue when compared to blue light [30]. It is proven in [31] that combination of both blue (415 + −5 nm, irradiance 40 mW/cm^2^, 48 J/cm^2^ ) and red (633 + − 6 nm, 80 mW/cm^2^ , 96 J/cm^2^) light produces an overall decrease in the melanin level. It is also shown that helium-neon laser irradiation with visible light significantly enhances the attachment of melanocytes to type IV collagen and stimulates migration and proliferation in melanocytes [32, 33]. This stimulates nerve growth factor which is a major paracrine maintenance factor responsible for melanocytes’ survival in the skin [34]. It is iterated in [35] that the green and blue light (488 and 514 nm, respectively) of the argon laser is especially absorbed by melanin which is produced by melanocytes [35].

In the classification scheme of this study, SVM [25] is used. The aim of the classification is to correctly classify dermoscopy images as malignant or non-malignant. The following parameters of SVM are varied to select the best classifier over others: SVM Type (ν-SVM, C-SVM), Kernel (Polynomial, Radial Basis, Linear), C (penalization parameter), R (coefficient), D (degree), and G (gamma parameter in the kernel function).

These parameters are determined after several experiments. We used three classification assessment methods in order to find the best set of features. These assessment methods are Leave-one-out, 10-fold cross validation, and Model accuracy. In terms of classification accuracy assessment methods, model accuracy, leave one out (LOO), and 10-fold cross validation (10CV) are standard methods in the machine learning and information gain related studies. Model Accuracy gives a sense that how the problem posed in the feature space (such as linearly separable, polynomial, or radial basis) and how we should approach to this problem. LOO and 10CV methods are similar to each other and assess how a classification model reacts to a new data point or dataset. The advantages of these methods are that they represent very significant information about the classification models we build. On the other hand, in LOO and 10CV assessments, we should leave out one or more than one sample from the original dataset so that they can be tested. This means that we should shrink the dataset to some extent. In the case of LOO, this issue is minimized because only one sample is removed from the dataset before the training of the classification model.

For our case, LOO uses 99 dermoscopy images for building a model and training the SVM classifier. Then, one of the dermoscopy images is used for testing purposes. In 10CV, 90 of the dermoscopy images are used for building a model and training the SVM classifier whereas 10 of the dermoscopy images are intended to use for testing the classifier. This process is repeated 10 times for different randomly selected 10 test data. In model accuracy, we use entire set of dermoscopy images for building a model and training purposes.

## Results and discussion

In the assessment of classification accuracy, we used standard metrics, precision, recall, and F-measure. Recall is the percentage of positive (malignant) labeled instances that were predicted as positive and found by TP/(TP + FN). Precision is defined as the percentage of positive predictions that are correct, and calculated by TP/(TP + FN). TP means true positives. In our case, TP corresponds to the melanoma lesions which are correctly classified as melanoma. TN means true negatives. TN corresponds to the non-melanoma lesions which are correctly classified as non-melanoma. FP means false positives. FP corresponds to the non-melanoma lesions which are incorrectly classified as melanoma. FN means false negatives. FN corresponds to the melanoma lesions which are incorrectly classified as non-melanoma.

F-measure is a harmonic mean of precision and recall and given as:

4.1 $$ \frac{2* \Pr ecision*\mathrm{R}\mathrm{e} call}{ \Pr ecision+\mathrm{R}\mathrm{e} call} $$

A high score of F-measure indicate that the classifier successfully finds targeted lesion without compromising precision and recall.

**Fig. 30:**
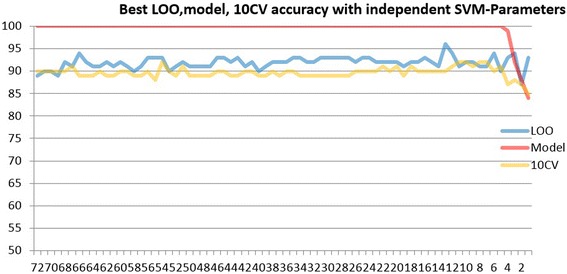
Experiments with only Blue, Cr, and Green-based features. The top (sorted best-to-worst) 10 features are: Gre-5-AH, Cr-10-AM, Gre-15-AM, Blu-10-MM, Blu-7-MH, Gre-15-MS, Gre-5-MH, Cr-7-AM, Blu-5-MS, and Blu-7-AS

The graph in Fig. 30 is shaped by using the best three features, which are Blue, Cr and Green-based features, each of them has 24 possible features and 3 color channels which makes a total of 72 features. As seen in the graph, 72 is the starting point for the x axis. This graph is obtained as follows. First, SVM classifier starts to run with these 72 features, and the best achieved accuracy is obtained and marked on the graph. In the next step, the worst feature is discarded from the 72 features, and 71 features are left behind to train and test SVM. The value which is obtained by this process is also marked on the graph which simply corresponds to 71 in the x axis. This discarding of one feature process is plotted until 1 feature is left behind in this routine. By doing so, the graph is formed and finalized as seen in Fig. 30.

Results presented in Fig. 30 are for the third method that we developed (the inner-shifted boundary by dynamic scaling, details provided in Section 3.4.5). The other two methods given in sections 3.4.3 (74 % accuracy) and 3.4.4 (83 %) as expected have not produced higher accuracies for malignancy detection. This is due to having noise (outside region of the lesion border) incorporated in these methods. Since the model accuracy is 100 %, this emphasizes that the proposed model for 3^rd^ method (section 3.4.5) can accurately distinguish malignant lesions from benign lesions. This means that the proposed model is an accurate approach for classification purposes.

**Fig. 31:**
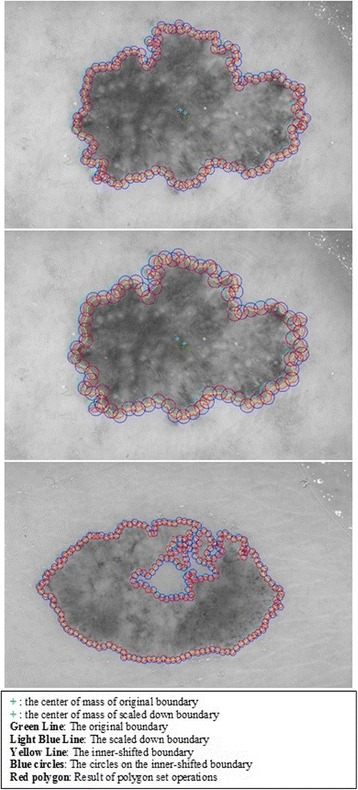
The intersecting *polygon circle* with (**c**) radius size 10 (**d**) radius size 15 (**e**) the fixed case

According to the graph in Fig. 30, it can be inferred that 13 features could yield optimal result. The feature size was determined through a supervised learning, which is a feature selection method with SVM RFE [26]. The subset with 13 features was selected from 240 candidates. This basically implies that 13 features are sufficient to model and train the SVM classifier. In the 13 feature scenario on Fig. 31, leave-one-out (LOO) methodology achieves up to 96 % classification accuracy and 10-fold cross validation (10CV) accomplishes up to 90 % classification accuracy. It should be noted that less than 6 features cannot represent the proposed model well based upon the graph.

**Fig. 32:**
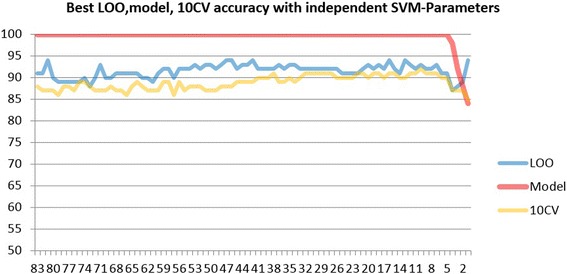
Experiments with only Blue, Cr, and Green-based homogeneity features and Fractal features. Top 25 features, where it seems all assessment models yield better cumulative: Gre-5-AH, Cr-10-AM, Blu-10-MM, Blu-5-AM, MassRadS, Blu-15-MS, Gre-5-MH, Cr-7-AM, Blu-7-MH, Blu-7-AM, Cr-15-AM, Blu-15-AH, Gre-7-AH, Blu-10-AS, Blu-5-MH, MassRadL, Blu-5-MS, Cr-15-AH, Cr-15-MH, Fast-Hyb, Blu-5-AH, CornerC, Cr-5-AM, Blu-15-AM, Gre-15-AH

In Fig. 32, the graph is formed by using the best three features plus fractal features. That means 83 total number of features are used for the experiments. The idea behind generating this graph is the same as one explained in the first case. In each training and testing cycle, the worst feature, based upon its ranking score, is eliminated from the 83 feature set, and training and testing is applied to the remaining feature and obtained classification accuracy is recorded and plotted on the graph. Again, this cycle routine is carried out until one feature is left behind. Thus, the graph is built up as illustrated in Fig. 32.

With the same analytical approach, as it is comprehended from the graph, 16 features stand out a promising result with 94 % correct classification accuracy for LOO method and 91 % correct classification accuracy for 10 CV. Less number of features also can be considered to represent the model.

## Conclusions

Accurate detection and objective evaluation of abrupt pigment pattern cutoff at the perimeter of a skin lesion is one of the important criteria for malignancy detection. However, it remains a challenging task and biased by a dermatologist’s experience level. In this article, a novel approach is proposed where abruptness of pigment pattern along the lesion perimeter is measured. The boundary of the skin lesion is determined with the help of the density based lesion border detection technique. By using two different methods, the detected boundary is step by step scaled down. Then, throughout scaled down borders, pigment motifs’ homogeneities are extracted and computed in various color channels and at different scales.

The proposed method has been experimented and validated by selecting a test bed which includes 100 dermoscopy images (69 benign and 31 melanoma). The results prove that the proposed approach is highly effective to detect malignancy in dermoscopy images. More specifically, up to 96 % classification accuracy, 0.96 specificity value, and 0.86 sensitivity value are accomplished in detection of malignancy in a particular color space. Finally, in addition to that, 0.87 of the F-measure is attained on malignancy detection.
